# A retrospective study on association between obesity and cardiovascular risk diseases with aging in Chinese adults

**DOI:** 10.1038/s41598-018-24161-0

**Published:** 2018-04-11

**Authors:** Jie Sun, Weihong Zhou, Tianwei Gu, Dalong Zhu, Yan Bi

**Affiliations:** 1 Department of Endocrinology, Drum Tower Hospital affiliated to Nanjing University Medical School, Nanjing, China; 2 Health Manager Center, Drum Tower Hospital affiliated to Nanjing University Medical School, Nanjing, China

## Abstract

This study aimed to investigate the prevalence of overweight and obesity and its relationship with cardiovascular risk diseases among different sex and age groups in an urban Chinese adult population. A retrospective analysis was performed on 384,061 Chinese adults aged 20 years and older in Nanjing. The age-standardized prevalence of overweight and obesity was 42.8% and 13.2% in men and 23.9% and 6.6% in women. A gradually increasing trend was observed in the prevalence of overweight and obesity from 2008 to 2016, especially in individuals aged 20~39 years. Overweight and obesity were significantly associated with increased risks of dyslipidemia, diabetes mellitus, hypertension, and hyperuricemia. Age weakened such relationship for both genders, which spiked in individuals aged 20~39 years. For men and women aged 20~39 years, the OR (95% CI) of obesity reached 4.23 (4.01–4.47) and 5.29 (4.63–6.04) for dyslipidemia, 3.70 (2.97–4.60) and 6.38 (3.86–10.55) for diabetes mellitus, 6.19 (5.76–6.64) and 9.36 (7.86–11.13) for hypertension, and 3.66 (3.45–3.88) and 6.65 (5.70–7.74) for hyperuricemia, respectively. The increasing trend in the epidemic of overweight and obesity is a risk factor for cardiovascular risk diseases in Chinese adults, especially in individuals aged 20~39 years.

## Introduction

Obesity has become a prevalent health problem because of its association with subsequent cardiovascular disease and all-cause mortality^1–3^. Its increasing prevalence is affecting a large proportion of individuals of all ages and races worldwide^4,5^. As a large developing country in the world, China has now joined the world epidemic of obesity with its rapid economic growth over the past three decades^6–8^. National data showed that the combined prevalence of overweight and obesity increased from 20.0% in 1992 to 29.9% in 2002^9^. The global data indicate a marked growth of obese population in China from 13th place for men and 10th place for women in 1975 to 1st place for both genders in 2014^8^. Although the prevalence of obesity is increasing rapidly in both urban and rural areas, it is significantly higher in urban than in rural population^10,11^. Accordingly, more attention should be paid to the epidemiological features of obesity in urban areas considering its great health burden.

Overweight and obesity are known to be associated with a number of cardiovascular risk diseases, such as hypertension, dyslipidemia, diabetes mellitus, and insulin resistance^12–15^. Besides, obesity is reported to be linked to hyperuricemia in recent cross-sectional studies^16,17^. However, despite such relationship discussed in previous studies, few have focused on age- and sex-specific association between obesity and cardiovascular risk diseases in urban areas. To date the influence of age on the relationship between obesity and cardiovascular risk diseases is unclear, and literature suggested that such association might become weaker or stronger with aging^18,19^. Therefore, a large retrospective study was conducted to investigate the prevalence of overweight and obesity and its relationship with cardiovascular risk diseases in different sex and age groups among Chinese urban adults in Nanjing.

## Results

### Prevalence rates for different BMI categories by sex and age groups

**Figure 1:**
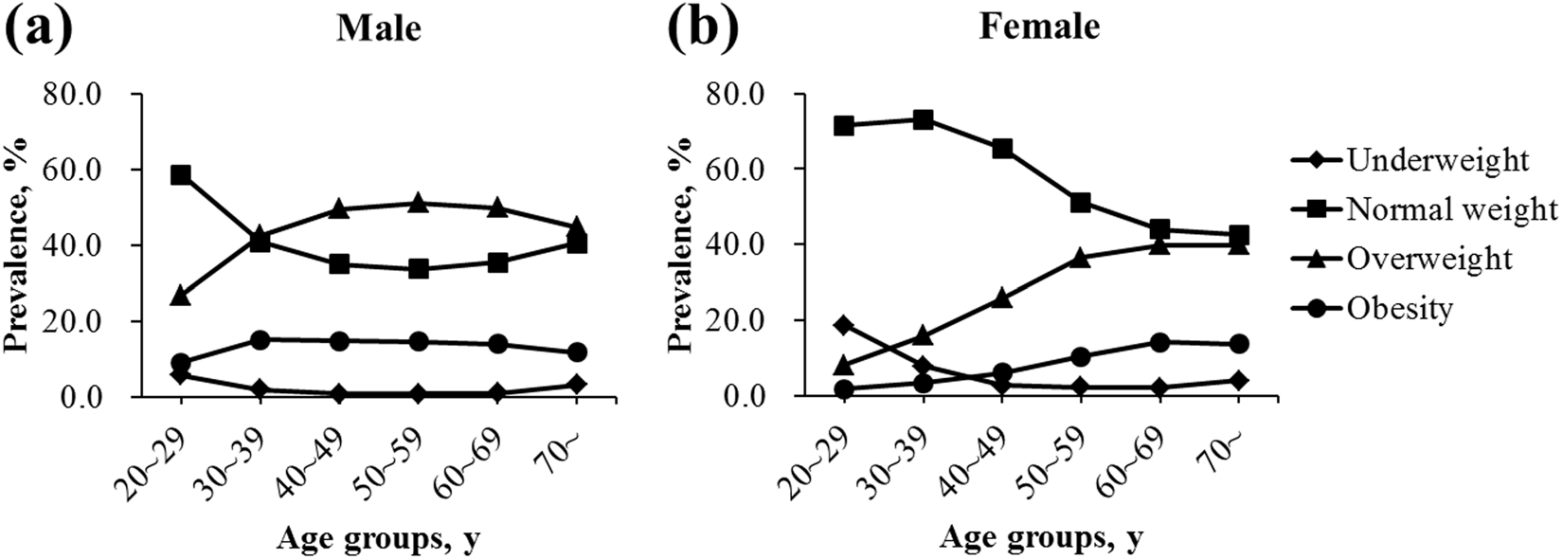
Prevalence rates of BMI categories by different age groups in (**a**) males and (**b**) females. BMI was categorized as underweight (<18.5 kg/m^2^), normal weight (18.5–23.9 kg/m^2^), overweight (24.0–27.9 kg/m^2^), and obese (≥28.0 kg/m^2^).

The mean values of BMI were 24.3 ± 3.3 and 22.0 ± 3.1 kg/m^2^ for men and women, respectively. The prevalence rates for different BMI categories by sex and age groups (20~29, 30~39, 40~49, 50~59, 60~69, and 70~ years) are shown in Fig. 1 (see Supplementary Table 1 for detailed information). For all ages, the age-standardized prevalence of overweight and obesity in males was higher compared with that in females (overweight: 42.8% vs 23.9%; obesity: 13.2% vs 6.6%). For different age groups, the prevalence of overweight in males was higher compared with that in females. Moreover, the prevalence of obesity in males was higher before 60 years of age but lower after 60 years compared with that in females. For men, a gradual increase was noted in the prevalence of overweight from the youngest age group (26.8%) up to the group aged 50~59 years (51.0%), followed by a decline in the higher age groups (60~69 and 70~ years). The prevalence of obesity was as high as about 15.0% between 30 and 60 years old, while declined after 60 years. For women, a steady increase was found in both the prevalence of overweight and obesity from the age group of 20~29 years (overweight: 8.2%; obesity: 1.7%) up to 60~69 years (overweight: 39.9%; obesity: 14.1%), followed by a decline in the highest age group of 70~ years. Notably, the prevalence of underweight was as high as 18.6% among young women aged 20~29 years and gradually decreased with the increase in age.

### Trends in the prevalence of overweight and obesity from 2008 to 2016

**Figure 2:**
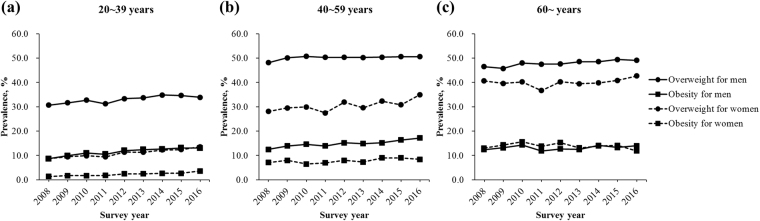
Prevalence rates of overweight and obesity between 2008 and 2016 in adults aged (**a**) 20~39 years, (**b**) 40~59 years, and (**c**) ≥60 years.

Trends in the prevalence of overweight and obesity by sex and age groups between 2008 and 2016 are demonstrated in Fig. 2. For all ages, the prevalence rate of overweight and obesity increased by 2.0% and 4.0% in males, and 4.3% and 1.5% in females from 2008 to 2016. For individuals aged 20~39 years, the prevalence of overweight and obesity increased slowly with the surveyed years. For men aged 40~59 years, a steady increase was found over time in the prevalence of obesity, while a stable level was observed in the prevalence of overweight. For women, there was no observable trend over time in the prevalence of overweight and obesity in the age group of 40~59 years. The prevalence rate peaked at 35.0% in 2016 for overweight and 9.0% in 2015 for obesity. For those aged ≥60 years, the prevalence of overweight showed a slow increasing trend from 2009 to 2015 in males, and from 2011 to 2016 in females. Nevertheless, no obvious trend over time in the prevalence of obesity was observed for both genders.

### Mean levels of metabolic markers by different BMI categories

The mean levels of metabolic markers by BMI categories and sex are displayed in Table 1. For both genders, the values of TG, LDL-c, fasting blood glucose, systolic pressure, diastolic pressure, and uric acid all increased significantly across the BMI categories (*P*_trend_ < 0.001), while the HDL-c level decreased with increasing BMI (*P*_trend_ < 0.001). Compared with women, significantly higher levels of TG, diastolic pressure, and uric acid as well as a significantly lower level of HDL-c were observed in males in all BMI categories (*P* < 0.001 for differences between sexes).

**Table 1:** Mean (95% CI) levels of metabolic markers for adults aged ≥20 years by different BMI categories and sex.

Metabolic markers	BMI categories				*P* *
	Underweight	Normal weight	Overweight	Obesity	
Male					
Triglyceride (mmol/L)	0.92 (0.91–0.94)	1.34 (1.33–1.34)	1.95 (1.94–1.96)	2.42 (2.39–2.44)	<0.001
High-density lipoprotein (mmol/L)	1.49 (1.47–1.51)	1.29 (1.28–1.29)	1.14 (1.13–1.14)	1.06 (1.05–1.06)	<0.001
Low-density lipoprotein (mmol/L)	2.12 (2.09–2.14)	2.44 (2.44–2.45)	2.61 (2.61–2.62)	2.66 (2.65–2.67)	<0.001
Fasting blood glucose (mmol/L)	4.95 (4.93–4.98)	5.20 (5.19–5.21)	5.48 (5.47–5.49)	5.69 (5.67–5.71)	<0.001
Systolic pressure (mmol/L)	113.01 (112.69–113.33)	118.88 (118.79–118.97)	126.07 (125.97–126.18)	131.80 (131.61–131.99)	<0.001
Diastolic pressure (mmol/L)	71.65 (71.43–71.88)	75.88 (75.81–75.94)	81.97 (81.89–82.05)	86.31 (86.16–86.45)	<0.001
Uric acid (mmol/L)	331.55 (328.95–334.15)	349.30 (348.72–349.87)	376.97 (376.38–377.56)	402.99 (401.85–404.13)	<0.001
Female					
Triglyceride (mmol/L)	0.77 (0.76–0.78)	0.99 (0.98–0.99)	1.50 (1.49–1.52)	1.76 (1.73–1.79)	<0.001
High-density lipoprotein (mmol/L)	1.64 (1.63–1.66)	1.51 (1.51–1.51)	1.35 (1.35–1.36)	1.27 (1.26–1.28)	<0.001
Low-density lipoprotein (mmol/L)	2.08 (2.07–2.10)	2.35 (2.34–2.36)	2.64 (2.63–2.65)	2.74 (2.72–2.76)	<0.001
Fasting blood glucose (mmol/L)	4.83 (4.82–4.84)	5.00 (5.00–5.01)	5.37 (5.35–5.38)	5.67 (5.64–5.71)	<0.001
Systolic pressure (mmol/L)	107.38 (107.21–107.55)	112.49 (112.41–112.58)	124.80 (124.60–125.01)	133.87 (133.45–134.29)	<0.001
Diastolic pressure (mmol/L)	68.90 (68.78–69.03)	71.32 (71.26–71.37)	77.84 (77.71–77.96)	82.94 (82.68–83.20)	<0.001
Uric acid (mmol/L)	250.89 (249.77–252.00)	258.74 (258.34–259.15)	287.28 (286.45–288.11)	313.94 (312.11–315.78)	<0.001

*Multiple linear regression with adjustment for age. Abbreviations: BMI, body mass index.

### Multivariable-adjusted odds ratios for cardiovascular risk diseases

**Figure 3:**
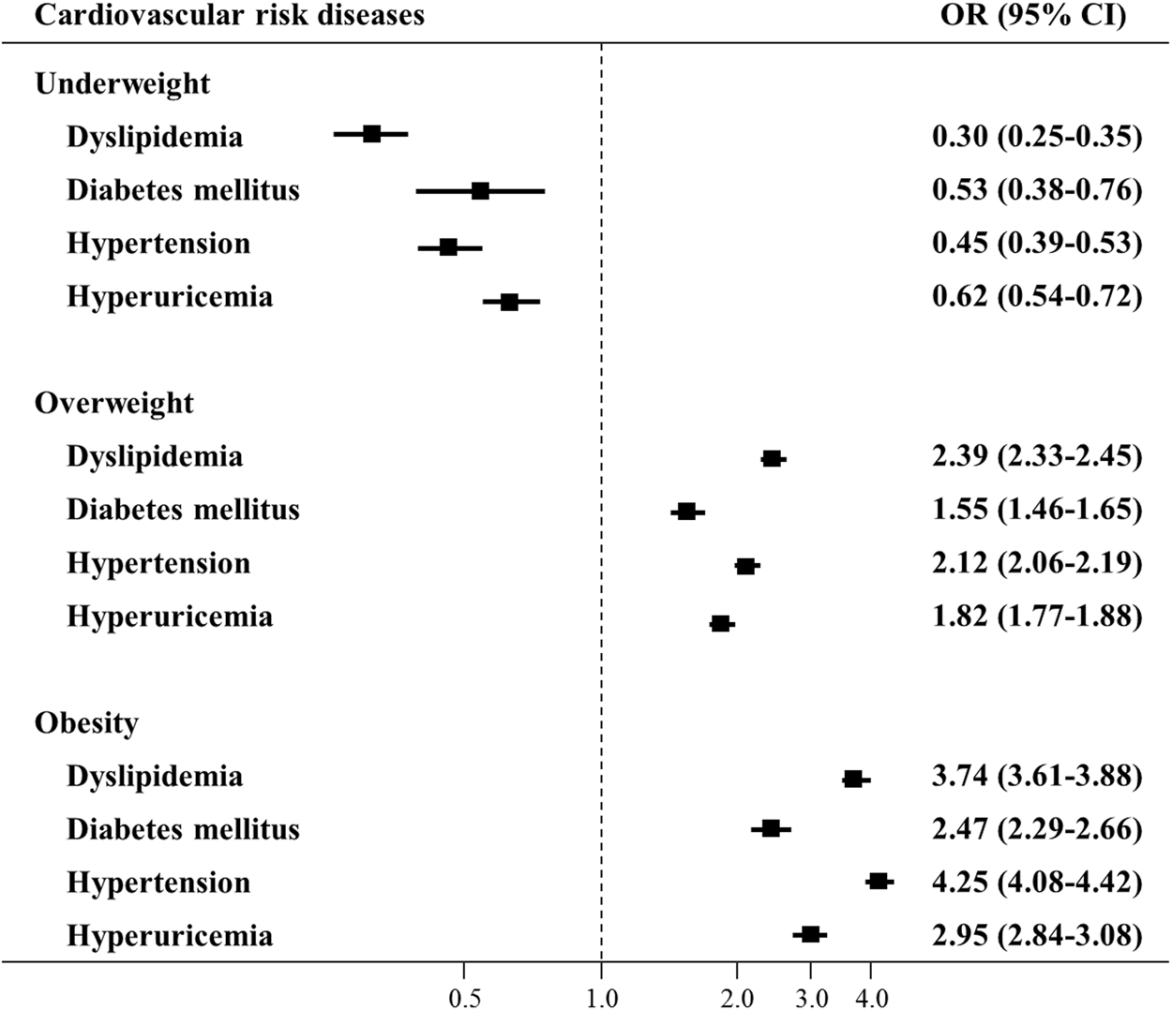
Relationship between different BMI categories and cardiovascular risk diseases among 215,536 males. Adjusted for age and other cardiovascular risk diseases. Squares represent the odds ratios (OR), and horizontal lines represent the corresponding 95% confidence intervals (CI).

**Figure 4:**
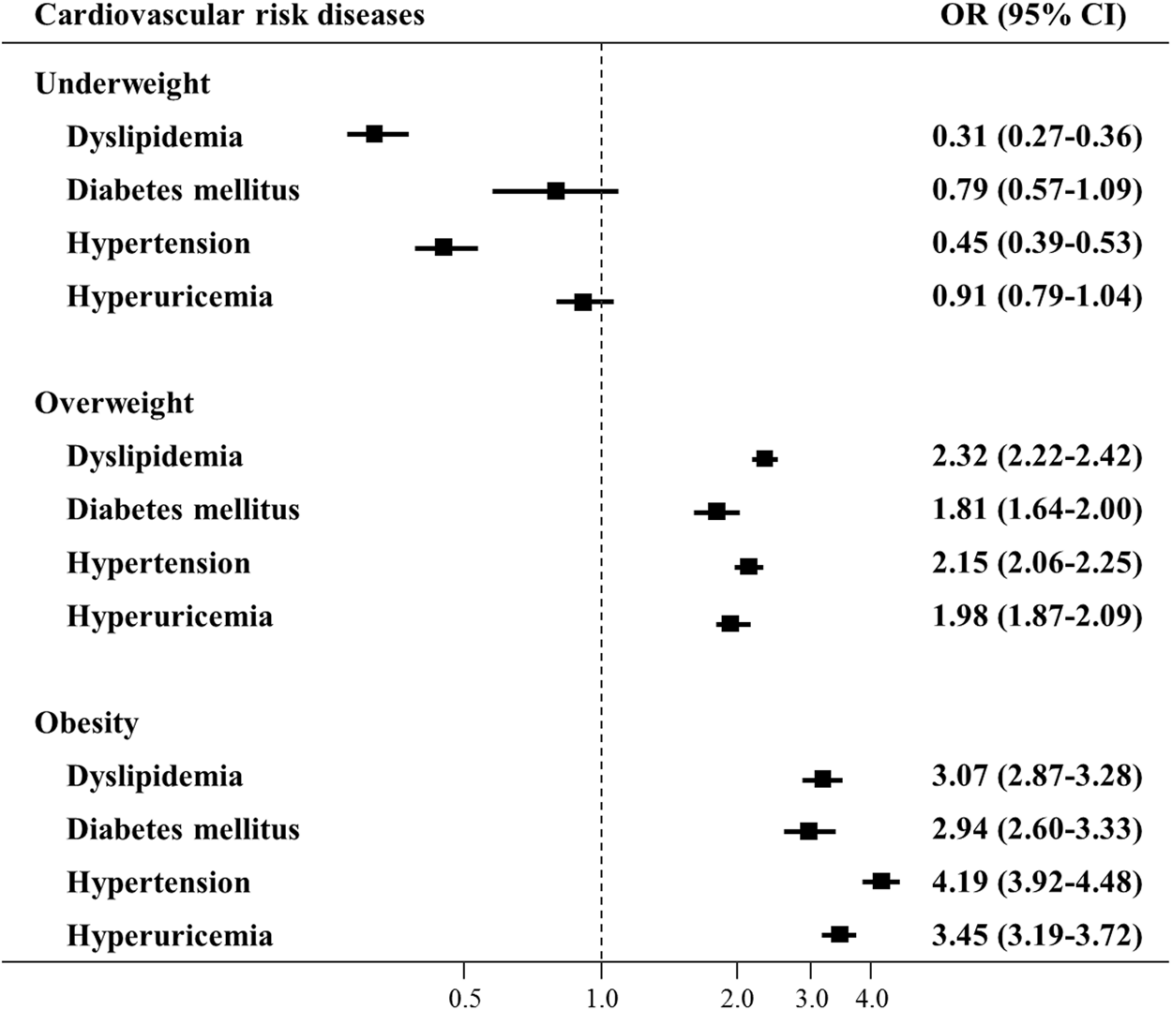
Relationship between different BMI categories and cardiovascular risk diseases among 168,525 females. Adjusted for age and other cardiovascular risk diseases. Squares represent the odds ratios (OR), and horizontal lines represent the corresponding 95% confidence intervals (CI).

Multivariate analyses of underweight, overweight, and obesity associated with cardiovascular risk diseases are shown in Fig. 3 for men and in Fig. 4 for women. Compared with people with normal weight, people with overweight and obesity had significantly increased risks of dyslipidemia, diabetes mellitus, hypertension, and hyperuricemia for both genders. For men, the OR (95% CI) of obesity reached 3.74 (3.61–3.88), 2.47 (2.29–2.66), 4.25 (4.08–4.42), and 2.95 (2.84–3.08), respectively. For women, the OR (95% CI) of obesity reached 3.07 (2.87–3.28), 2.94 (2.60–3.33), 4.19 (3.92–4.48), and 3.45 (3.19–3.72), respectively. On the contrary, people with underweight were at lower risks of cardiovascular risk diseases.

**Figure 5:**
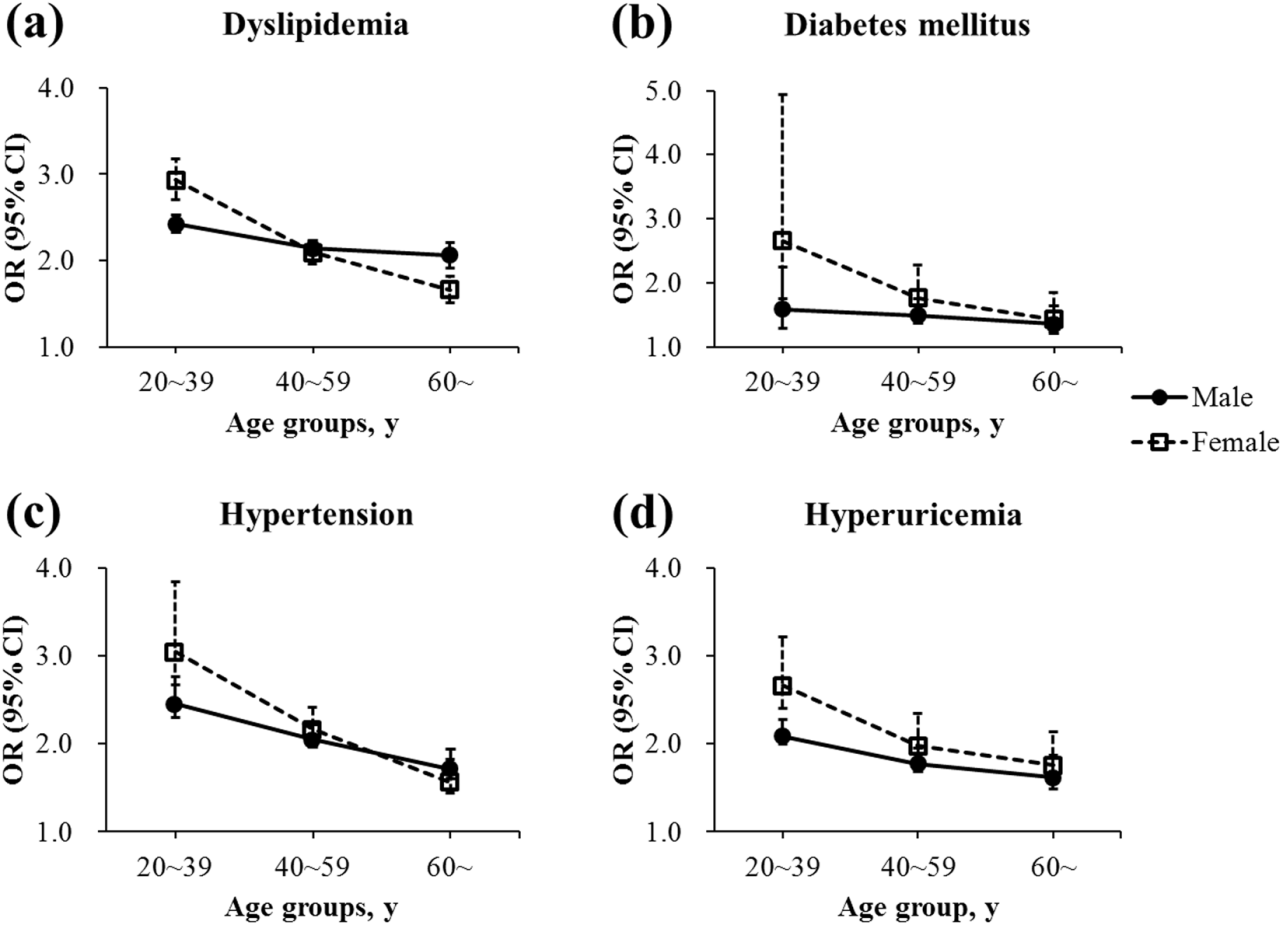
Relationship between overweight and (**a**) dyslipidemia, (**b**) diabetes mellitus, (**c**) hypertension, and (**d**) hyperuricemia by sex and age groups. Adjusted for age and other cardiovascular risk diseases. Squares and dots represent the odds ratios (OR), and vertical lines represent the corresponding 95% confidence intervals (CI).

**Figure 6:**
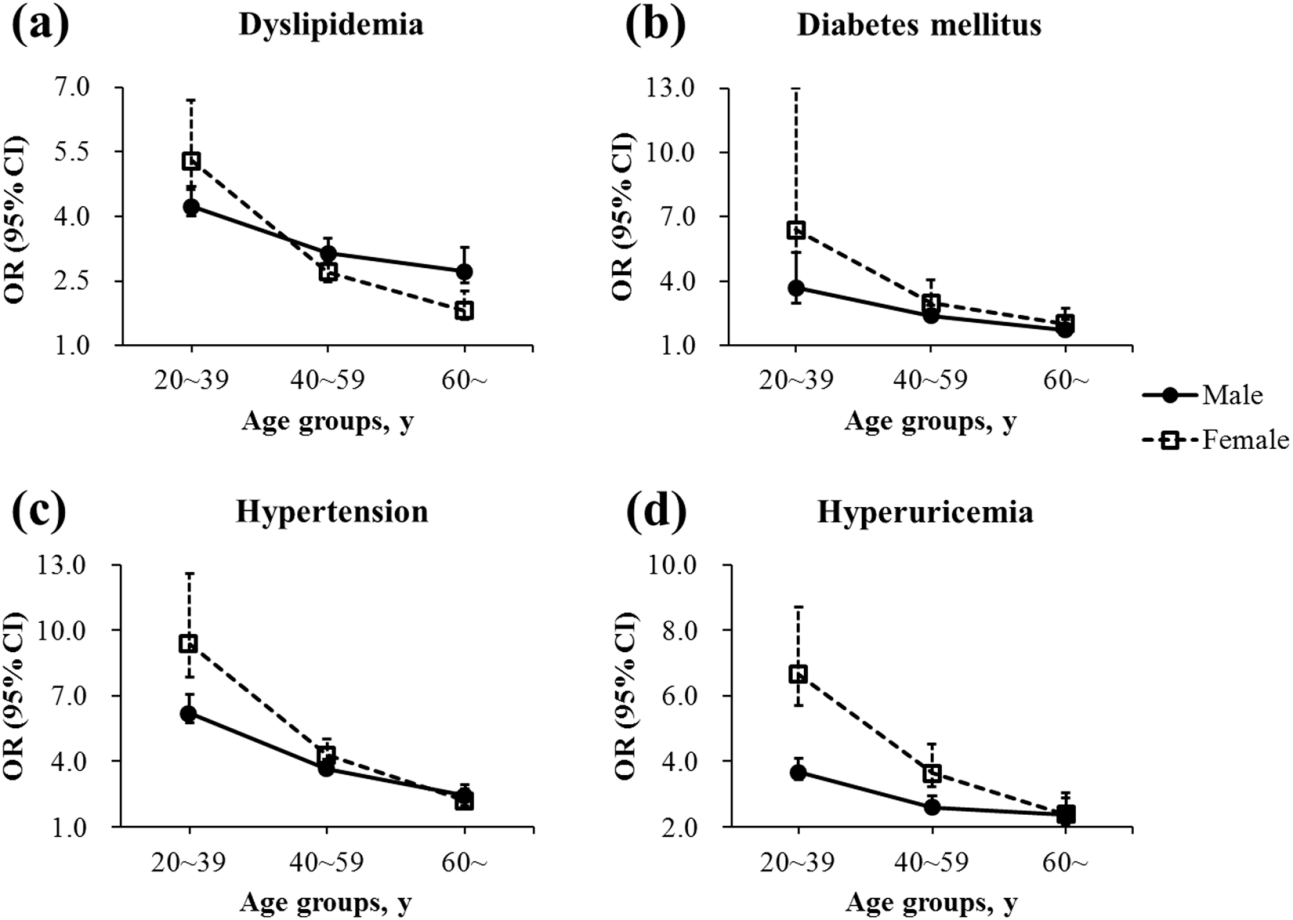
Relationship between obesity and (**a**) dyslipidemia, (**b**) diabetes mellitus, (**c**) hypertension, and (**d**) hyperuricemia by sex and age groups. Adjusted for age and other cardiovascular risk diseases. Squares and dots represent the odds ratios (OR), and vertical lines represent the corresponding 95% confidence intervals (CI).

Age- and sex-specific associations of overweight and obesity with cardiovascular risk diseases were further analyzed and illustrated in Fig. 5 and in Fig. 6, respectively. Detailed information was shown in Supplementary Table 2 and Supplementary Table 3. For both overweight and obesity, age weakened its relationship with dyslipidemia, diabetes mellitus, hypertension, and hyperuricemia for both genders, which spiked in individuals aged 20~39 years. For men and women aged 20~39 years, the OR (95% CI) of obesity reached 4.23 (4.01–4.47) and 5.29 (4.63–6.04) for dyslipidemia, 3.70 (2.97–4.60) and 6.38 (3.86–10.55) for diabetes mellitus, 6.19 (5.76–6.64) and 9.36 (7.86–11.13) for hypertension, and 3.66 (3.45–3.88) and 6.65 (5.70–7.74) for hyperuricemia, respectively.

## Discussion

The present study provided the detailed epidemiological features of overweight and obesity in Chinese urban areas, which are strong predictors of cardiovascular risk diseases, especially in individuals aged 20~39 years.

Standardized by Chinese census data, 42.8% of men and 23.9% of women were overweight and a further 13.2% of men and 6.6% of women were obese in the analyzed population, which was similar to the findings of a previous large-scale survey among Chinese urban population^20^. Globally, the prevalence of overweight and obesity in China is lower than that in many European countries^1^; however, it ranks first in the number of obese individuals for both genders due to its huge population base^8^. Nonetheless, a gradually narrowing gap is observed in the prevalence of obesity between China and Western countries^21^. The present study indicated that the prevalence of overweight and obesity increased over time from 2008 to 2016, especially in individuals aged 20~39 years (Fig. 2), and this trend was consistent with those of the former studies in China^22–24^. With the rapid development of economy, lifestyles and nutrition, that is, improved food supplies, expansion of computerization, enhanced car ownership, rise of sedentary jobs, and so forth, have changed dramatically and contributed to the increase in the prevalence of overweight and obesity in China in recent years. Importantly, young adults are more susceptible to these unhealthy diets and sedentary lifestyles and pay less attention to their health compared with the middle-aged and elderly people, which might be a reason for the more obvious upward trend of overweight and obesity in the age group of 20~39 years. As the most populous country in the world, even 1.0% growth in the prevalence means an increase of nearly 14,000,000 obese people in China. It is a great health burden that China is suffering from. Thus, prevention and control of obesity should be the top priority in public health in China, with a focus on individuals aged 20~39 years.

Gender differences were found in this study in terms of overweight and obesity, and the prevalence was higher in males than in females before 60 years of age. It was consistent with an earlier report on Chinese population^20^. While women usually do more housework and are more likely to control body weight because of concern for their figures, men may consume more high-fat dishes and alcohol due to frequent social gatherings, leading to such a difference. Furthermore, this study demonstrated a higher prevalence of obesity in women than in men after 60 years of age, which might be a result of changes in the secretion of hormones in postmenopausal women^25,26^. Women generally tend to gain weight after menopause due to the redistribution of body fat and the loss of muscle mass and strength, accompanied by hormonal changes^27^. Consistent with previous studies performed in China^28,29^, other Asian countries^30,31^, and the United States^32^ this study revealed that the prevalence of overweight and obesity increased with age but started to decline at 70 years in women and at 60 years in men. This decline at older ages may be partly due to the onset of sarcopenia in elderly populations^33^, the obesity-related mortality^34^, and cohort effect. Therefore, policymakers and experts should not only make a general rule to prevent overweight and obesity but also develop sex- and age-specific ones.

A variety of population studies have clearly established that overweight and obesity are associated with cardiovascular risk diseases^35–38^. However, the influence of age on such relationship remains unclear. The present study disclosed that obese individuals aged 20~39 years were at highest risks of cardiovascular risk diseases (Figs 5 and 6). The behavioral characteristics of younger adults, including more fatty food intake, less physical activity, shorter sleeping hours, more daily hassles, and poorer medication adherence, all contributed to the high occurrence of cardiovascular risk diseases. Moreover, the strong relationship between overweight and obesity and cardiovascular risk diseases was further demonstrated to be attenuated with increasing age (Figs 5 and 6), in consonance with the previous literature^39,40^. As people grow older, their physical function gradually degenerates^41^, whereas the cumulative effects of various risk factors aggravate, resulting in reduced contributions of overweight and obesity to the development of dyslipidemia, diabetes, hypertension, and hyperuricemia. Another potential hypothesis could be the so-called selective survival effect^42^ of older but healthier individuals, which indicates a possibility that unhealthy individuals with a high BMI might have already died from the adverse effects of obesity. Older people are at an increased risk for morbidity and mortality^42^ that is unrelated to obesity. Considering the findings that the prevalence of overweight and obesity also increased over time in the same age group of 20~39 years (Fig. 2a), a rising trend of cardiovascular diseases could occur among working-age adults, reinforcing the urgency to prevent and control obesity especially in the youth, and consequently reducing the health burden of obesity-related cardiovascular diseases in China in the coming decades^43,44^.

The main strengths of this study included its markedly large sample size and age- and sex-specific analysis on the association between overweight and obesity and cardiovascular risk diseases. However, some limitations should be noted. First of all, given the inherent flaws of cross-sectional studies, the cohort effect on the prevalence of overweight and obesity could not be evaluated in the present study. Causal inferences on the relationship between overweight and obesity and cardiovascular risk diseases could not be made. Second, all participants involved were from a single examination center, and a selection bias was inevitable when extrapolating the results to the general population. The age- and sex-standardized prevalence of overweight and obesity was calculated using Chinese census data to enhance the representativeness of the present study. Third, a small percentage of latent autoimmune diabetes in adults (LADA) could not be excluded because the islet autoantibodies of patients were not measured in this study. However, according to LADA China Study^45^, the prevalence of LADA in Chinese adults is no more than 6.0%. Additionally, when collecting medical history of the participants, we have excluded previously diagnosed type 1 diabetes. Finally, some residual confounding might exist because information on smoking status, physical activity, and so forth, was not collected, which could act as potential mediators for the relationship between obesity and cardiovascular risk diseases.

In conclusion, the present study showed a high prevalence of overweight and obesity in Chinese adults, which increased over time, especially in individuals aged 20~39 years. Moreover, overweight and obesity are risk factors for cardiovascular risk diseases in Chinese adults, especially in those aged 20~39 years. Emphasis should be placed on the youth in relation to the prevention and control of obesity. Further longitudinal studies in diverse populations are warranted to confirm these findings and enhance their generalizability.

## Methods

### Study design and population

In this study, 384,061 subjects (men, 215,536; women, 168,525) aged 20 years and above who were self-referred for a routine health check-up at the medical examination center of Nanjing Drum Tower Hospital (Nanjing, China) were retrospectively reviewed from January 2008 to July 2016. Information on age, gender, and body mass index (BMI) were available for all subjects. Serum triglyceride (TG), high-density lipoprotein (HDL-c), and low-density lipoprotein (LDL-c) levels were measured in 271,354 (70.7%), 163,461 (42.6%), and 163,446 (42.6%) individuals, respectively. Blood pressure and fasting glucose levels were measured in 74.7% (*n* = 286,998) and 99.9% (*n* = 384,013) of all subjects. Serum uric acid levels were measured in a total of 253,991 (66.1%) individuals. This study was approved by the ethics review committee of Nanjing Drum Tower Hospital, and all experimental protocols were in accordance with guidelines approved by the Institutional Review Board for Human Studies of Nanjing Drum Tower Hospital. A written informed consent was obtained from each subject.

### Measurement and definition of the variables

Weight, height, and blood pressure were measured using standard instruments and protocols by medical staff. Biochemical markers were measured using the blood samples of overnight fasted subjects in the central laboratory of Nanjing Drum Tower Hospital, which was authorized to perform laboratory tests according to the international quality standard ISO15189. BMI was calculated as weight in kilograms divided by the height in meters squared. According to the Chinese criteria of the Working Group on Obesity in China^46^, BMI was categorized as underweight (<18.5 kg/m^2^), normal weight (18.5–23.9 kg/m^2^), overweight (24.0–27.9 kg/m^2^), and obese (≥28.0 kg/m^2^). Prevalent hypertension was defined as a physician diagnosis of hypertension, measured systolic blood pressure ≥140 mm Hg or diastolic blood pressure ≥90 mm Hg, or use of antihypertensive medications. Definite causes of secondary hypertension were excluded, including pituitary diseases, thyroid disease, adrenal diseases, and kidney disease. Prevalent diabetes was defined as a physician diagnosis of type 2 diabetes, measured fasting blood glucose ≥7.0 mmol/L, or use of blood glucose regulator medications. Patients diagnosed with type 1 diabetes were excluded. Dyslipidemia was defined as a physician diagnosis of dyslipidemia, measured TG ≥ 2.26 mmol/L, LDL-c ≥ 4.14 mmol/L, or HDL-c < 1.04 mmol/L, or use of dyslipidemia medications. Hyperuricemia was defined as a physician diagnosis of hyperuricemia, measured uric acid >420 µmol/L in men and >360 µmol/L in women, or use of uric acid regulator medications.

### Statistical analysis

For people with multiple visits to the examination center, only the records of their first visits were kept for current analysis. Prevalence estimates for different BMI categories were calculated for subgroups according to age group and sex. Age- and sex-standardized prevalence rates were calculated by the direct method using Chinese census data in 2010. Mean values and 95% confidence intervals for metabolic markers were determined according to sex and BMI categories. Tests for linear trend across BMI categories were performed using the multiple linear regression method with adjustment for age. Multivariate logistic regression analyses were used to assess the associations of underweight, overweight, and obesity with prevalent cardiovascular risk diseases. The odds ratios and 95% confidence intervals were given. Data analyses were carried out using the R software (version 3.0.2, 2013-09-25; R Foundation for Statistical Computing, http://www.cran.r-project.org/). The significance level was set at *P* < 0.05, and the *P* values were given for two-sided tests.

### Data Availability Statement

The datasets generated during the current study are available from the corresponding author on reasonable request.

## Electronic supplementary material

Supplementary Table 1, 2, and 3

## References

[1] Collaborators GBDO (2017). Health Effects of Overweight and Obesity in 195 Countries over 25 Years. N. Engl. J. Med..

[2] Flegal KM, Kit BK, Orpana H, Graubard BI (2013). Association of all-cause mortality with overweight and obesity using standard body mass index categories: a systematic review and meta-analysis. JAMA.

[3] Prospective Studies C (2009). Body-mass index and cause-specific mortality in 900 000 adults: collaborative analyses of 57 prospective studies. Lancet.

[4] Kelly T, Yang W, Chen CS, Reynolds K, He J (2008). Global burden of obesity in 2005 and projections to 2030. Int J Obes (Lond).

[5] Ng M (2014). Global, regional, and national prevalence of overweight and obesity in children and adults during 1980–2013: a systematic analysis for the Global Burden of Disease Study 2013. Lancet.

[6] Chen CM (2008). Overview of obesity in Mainland China. Obes Rev.

[7] Cheng TO (2014). China’s epidemic of child obesity: an ounce of prevention is better than a pound of treatment. Int J Cardiol.

[8] Collaboration NCDRF (2016). Trends in adult body-mass index in 200 countries from 1975 to 2014: a pooled analysis of 1698 population-based measurement studies with 19.2 million participants. Lancet.

[9] Wang Y, Mi J, Shan XY, Wang QJ, Ge KY (2007). Is China facing an obesity epidemic and the consequences? The trends in obesity and chronic disease in China. Int J Obes (Lond).

[10] Zhang YX, Wang ZX, Zhao JS, Chu ZH (2016). Prevalence of Overweight and Obesity among Children and Adolescents in Shandong, China: Urban-Rural Disparity. J Trop Pediatr.

[11] Wan Y (2016). Body Mass Index of Young Men in China: Results From Four National Surveys Conducted Between 1955 and 2012. Medicine (Baltimore).

[12] Cameron AJ (2008). Central obesity as a precursor to the metabolic syndrome in the AusDiab study and Mauritius. Obesity (Silver Spring).

[13] Srinivasan SR (2009). Utility of waist-to-height ratio in detecting central obesity and related adverse cardiovascular risk profile among normal weight younger adults (from the Bogalusa Heart Study). Am J Cardiol.

[14] Hsieh SD, Muto T (2006). Metabolic syndrome in Japanese men and women with special reference to the anthropometric criteria for the assessment of obesity: Proposal to use the waist-to-height ratio. Prev Med.

[15] Hsieh SD, Muto T (2005). The superiority of waist-to-height ratio as an anthropometric index to evaluate clustering of coronary risk factors among non-obese men and women. Prev Med.

[16] Zhang N (2016). A Body Shape Index and Body Roundness Index: Two new body indices for detecting association between obesity and hyperuricemia in rural area of China. Eur J Intern Med.

[17] Duan Y (2015). Association between serum uric acid levels and obesity among university students (China). Nutr Hosp.

[18] Vyssoulis G (2011). Effect of age on interdependence and hierarchy of cardiovascular risk factors in hypertensive patients. Am J Cardiol.

[19] Alexander CM, Landsman PB, Grundy SM (2008). The influence of age and body mass index on the metabolic syndrome and its components. Diabetes Obes Metab.

[20] Xu T, Zhu G, Han S (2015). Trend of Body Compositions with Aging among Chinese Adolescents, Adults and Elders. J Nutr Health Aging.

[21] Jin MJ (2013). Prevalence of overweight and obesity and their associations with socioeconomic status in a rural Han Chinese adult population. PLoS One.

[22] Xi B (2012). Secular trends in the prevalence of general and abdominal obesity among Chinese adults, 1993–2009. Obes Rev.

[23] Wildman RP (2008). Trends in overweight and obesity in Chinese adults: between 1991 and 1999–2000. Obesity (Silver Spring).

[24] Wang H, Du S, Zhai F, Popkin BM (2007). Trends in the distribution of body mass index among Chinese adults, aged 20–45 years (1989-2000). Int J Obes (Lond).

[25] Maltais ML, Desroches J, Dionne IJ (2009). Changes in muscle mass and strength after menopause. J Musculoskelet Neuronal Interact.

[26] Kaji H (2014). Interaction between Muscle and Bone. J Bone Metab.

[27] Crawford SL, Casey VA, Avis NE, McKinlay SM (2000). A longitudinal study of weight and the menopause transition: results from the Massachusetts Women’s Health Study. Menopause.

[28] Reynolds K (2007). Prevalence and risk factors of overweight and obesity in China. Obesity (Silver Spring).

[29] Gu D (2005). Prevalence of the metabolic syndrome and overweight among adults in China. Lancet.

[30] Ismail MN (2002). Obesity in Malaysia. Obes Rev.

[31] Moon OR, Kim NS, Jang SM, Yoon TH, Kim SO (2002). The relationship between body mass index and the prevalence of obesity-related diseases based on the 1995 National Health Interview Survey in Korea. Obes Rev.

[32] An R, Xiang X (2016). Age-period-cohort analyses of obesity prevalence in US adults. Public Health.

[33] Rizzoli R (2013). Quality of life in sarcopenia and frailty. Calcif Tissue Int.

[34] McGee DL (2005). & Diverse Populations, C. Body mass index and mortality: a meta-analysis based on person-level data from twenty-six observational studies. Ann Epidemiol.

[35] Dankel SJ, Loenneke JP, Loprinzi PD (2015). The impact of overweight/obesity duration on the association between physical activity and cardiovascular disease risk: an application of the “fat but fit” paradigm. Int J Cardiol.

[36] Roberts VH, Frias AE, Grove KL (2015). Impact of maternal obesity on fetal programming of cardiovascular disease. Physiology (Bethesda).

[37] Lee SY (2014). The impact of obesity on subclinical coronary atherosclerosis according to the risk of cardiovascular disease. Obesity (Silver Spring).

[38] Li M, McDermott RA (2010). Using anthropometric indices to predict cardio-metabolic risk factors in Australian indigenous populations. Diabetes Res Clin Pract.

[39] Nanas S (1987). The role of relative weight in the positive association between age and serum cholesterol in men and women. J Chronic Dis.

[40] Canning KL, Brown RE, Jamnik VK, Kuk JL (2014). Relationship between obesity and obesity-related morbidities weakens with aging. J Gerontol A Biol Sci Med Sci.

[41] Terman A (2006). Catabolic insufficiency and aging. Ann N Y Acad Sci.

[42] Janssen I, Mark AE (2007). Elevated body mass index and mortality risk in the elderly. Obes Rev.

[43] Murray CJ (2012). Disability-adjusted life years (DALYs) for 291 diseases and injuries in 21 regions, 1990–2010: a systematic analysis for the Global Burden of Disease Study 2010. Lancet.

[44] Kankeu HT, Saksena P, Xu K, Evans DB (2013). The financial burden from non-communicable diseases in low- and middle-income countries: a literature review. Health Res Policy Syst.

[45] Zhou Z (2013). Frequency, immunogenetics, and clinical characteristics of latent autoimmune diabetes in China (LADA China study): a nationwide, multicenter, clinic-based cross-sectional study. Diabetes.

[46] Zhou BF (2002). & Cooperative Meta-Analysis Group of the Working Group on Obesity in, C. Predictive values of body mass index and waist circumference for risk factors of certain related diseases in Chinese adults–study on optimal cut-off points of body mass index and waist circumference in Chinese adults. Biomed Environ Sci.

