# Supplementary material for: A retrospective study on association between obesity and cardiovascular risk diseases with aging in Chinese adults
Source: Sci Rep. 2018 Apr 11;8:5806. doi: 10.1038/s41598-018-24161-0 (PMC5895579; doi:10.1038/s41598-018-24161-0)
Supplement: Supplementary file 1 — Supplementary Table 1, 2, and 3 [file 41598_2018_24161_MOESM1_ESM.doc]

**A retrospective study on association between obesity and cardiovascular risk diseases with aging in Chinese adults**

**Jie Sun^1†^, Weihong Zhou^2†^, Tianwei Gu^1†^, Dalong Zhu^1^ and Yan Bi^1*^**

^1^ Department of Endocrinology, Drum Tower Hospital affiliated to Nanjing University Medical School, Nanjing, China

^2^ Health Manager Center, Drum Tower Hospital affiliated to Nanjing University Medical School, Nanjing, China

**^†^** Jie Sun, Weihong Zhou, and Tianwei Gu contributed equally to the manuscript.

* Corresponding author:

Yan Bi: Department of Endocrinology, Drum Tower Hospital affiliated to Nanjing University Medical School, No. 321 Zhongshan Road, Nanjing, 210008, China; Tel (Fax): 86-25-68182474; E-mail: biyan@nju.edu.cn.

**Supplementary Table 1. Prevalence (95% CI) of BMI categories for adults aged ≥20 years by sex and age groups**

| **Age groups (years)** | **BMI categories** | | | | | | | |
| --- | --- | --- | --- | --- | --- | --- | --- | --- |
|  | ***n*** | **Underweight (%)** | ***n*** | **Normal weight (%)** | ***n*** | **Overweight (%)** | ***n*** | **Obesity (%)** |
| Male |  |  |  |  |  |  |  |  |
| 20~29 | 4,604 | 5.70 (5.54–5.86) | 47,306 | 58.54 (58.20–58.88) | 21,642 | 26.78 (26.48–27.09) | 7,260 | 8.98 (8.79–9.18) |
| 30~39 | 929 | 1.83 (1.72–1.95) | 20,636 | 40.71 (40.28–41.13) | 21,528 | 42.46 (42.03–42.90) | 7,603 | 15.00 (14.69–15.31) |
| 40~49 | 311 | 0.84 (0.75–0.93) | 12,993 | 34.94 (34.46–35.43) | 18,394 | 49.47 (48.96–49.98) | 5,486 | 14.75 (14.39–15.12) |
| 50~59 | 213 | 0.78 (0.68–0.89) | 9,219 | 33.65 (33.09–34.22) | 13,973 | 51.01 (50.41–51.60) | 3,989 | 14.56 (14.15–14.98) |
| 60~69 | 99 | 0.87 (0.71–1.06) | 4,025 | 35.38 (34.50–36.26) | 5,659 | 49.74 (48.81–50.66) | 1,595 | 14.02 (13.39–14.67) |
| 70~ | 252 | 3.12 (2.75–3.52) | 3,263 | 40.42 (39.35–41.50) | 3,612 | 44.75 (43.66–45.84) | 945 | 11.71 (11.01–12.43) |
| Total | 6,408 | 2.97 (2.90–3.05) | 97,442 | 45.21 (45.00–45.42) | 84,808 | 39.35 (39.14–39.55) | 26,878 | 12.47 (12.33–12.61) |
| Age-standardized |  | 2.30 (2.16–2.45) |  | 41.72 (41.19–42.25) |  | 42.79 (42.25–43.34) |  | 13.19 (12.82–13.57) |
| Female |  |  |  |  |  |  |  |  |
| 20~29 | 13,543 | 18.57 (18.29–18.85) | 52,220 | 71.60 (71.28–71.93) | 5,945 | 8.15 (7.95–8.35) | 1,220 | 1.67 (1.58–1.77) |
| 30~39 | 2,899 | 7.73 (7.47–8.01) | 27,402 | 73.11 (72.66–73.56) | 5,961 | 15.90 (15.54–16.28) | 1,218 | 3.25 (3.07–3.43) |
| 40~49 | 722 | 2.75 (2.55–2.95) | 17,207 | 65.46 (64.88–66.03) | 6,766 | 25.74 (25.21–26.27) | 1,593 | 6.06 (5.77–6.36) |
| 50~59 | 399 | 2.13 (1.93–2.35) | 9,580 | 51.10 (50.38–51.82) | 6,845 | 36.51 (35.82–37.21) | 1,923 | 10.26 (9.83–10.70) |
| 60~69 | 155 | 1.98 (1.68–2.31) | 3,451 | 44.00 (42.89–45.10) | 3,130 | 39.90 (38.82–41.00) | 1,108 | 14.13 (13.36–14.92) |
| 70~ | 206 | 3.93 (3.42–4.50) | 2,229 | 42.55 (41.21–43.91) | 2,088 | 39.86 (38.53–41.20) | 715 | 13.65 (12.73–14.61) |
| Total | 17,924 | 10.64 (10.49–10.78) | 112,089 | 66.51 (66.29–66.74) | 30,735 | 18.24 (18.05–18.42) | 7,777 | 4.61 (4.52–4.72) |
| Age-standardized |  | 7.31 (7.04–7.59) |  | 62.21 (61.58–62.84) |  | 23.93 (23.37–24.50) |  | 6.55 (6.21–6.91) |

Abbreviations: BMI, body mass index.

**Supplementary Table 2. Age- and sex-specific associations of overweight with cardiovascular risk diseases. Values are odds ratio (95% confidence intervals)**

| **Age groups (years)** | **Cardiovascular risk diseases** | | | |
| --- | --- | --- | --- | --- |
|  | **Dyslipidemia** | **Diabetes mellitus** | **Hypertension** | **Hyperuricemia** |
| Male |  |  |  |  |
| 20~39 | 2.42 (2.33–2.52) | 1.59 (1.29–1.95) | 2.45 (2.30–2.61) | 2.08 (1.99–2.18) |
| 40~59 | 2.14 (2.06–2.23) | 1.50 (1.37–1.63) | 2.04 (1.96–2.13) | 1.77 (1.68–1.86) |
| 60~ | 2.06 (1.91–2.21) | 1.36 (1.22–1.50) | 1.71 (1.60–1.83) | 1.61 (1.48–1.74) |
| *P* for interaction | <0.001 | <0.001 | <0.001 | <0.001 |
| Female |  |  |  |  |
| 20~39 | 2.93 (2.70–3.17) | 2.66 (1.76–4.03) | 3.04 (2.67–3.47) | 2.66 (2.40–2.95) |
| 40~59 | 2.09 (1.96–2.22) | 1.77 (1.53–2.04) | 2.16 (2.04–2.29) | 1.97 (1.80–2.17) |
| 60~ | 1.66 (1.51–1.82) | 1.43 (1.24–1.66) | 1.56 (1.44–1.70) | 1.75 (1.57–1.95) |
| *P* for interaction | <0.001 | <0.001 | <0.001 | 0.922 |

Adjusted for age and other cardiovascular risk diseases.

**Supplementary Table 3. Age- and sex-specific associations of obesity with cardiovascular risk diseases. Values are odds ratio (95% confidence intervals)**

| **Age groups (years)** | **Cardiovascular risk diseases** | | | |
| --- | --- | --- | --- | --- |
|  | **Dyslipidemia** | **Diabetes mellitus** | **Hypertension** | **Hyperuricemia** |
| Male |  |  |  |  |
| 20~39 | 4.23 (4.01–4.47) | 3.70 (2.97–4.60) | 6.19 (5.76–6.64) | 3.66 (3.45–3.88) |
| 40~59 | 3.14 (2.97–3.32) | 2.39 (2.16–2.64) | 3.67 (3.47–3.89) | 2.60 (2.44–2.78) |
| 60~ | 2.72 (2.46–3.02) | 1.72 (1.50–1.98) | 2.44 (2.21–2.70) | 2.36 (2.11–2.64) |
| *P* for interaction | <0.001 | <0.001 | <0.001 | <0.001 |
| Female |  |  |  |  |
| 20~39 | 5.29 (4.63–6.04) | 6.38 (3.86–10.55) | 9.36 (7.86–11.13) | 6.65 (5.70–7.74) |
| 40~59 | 2.71 (2.47–2.98) | 2.99 (2.50–3.57) | 4.28 (3.92–4.67) | 3.63 (3.21–4.10) |
| 60~ | 1.81 (1.60–2.05) | 2.01 (1.68–2.40) | 2.21 (1.96–2.49) | 2.38 (2.07–2.73) |
| *P* for interaction | <0.001 | <0.001 | <0.001 | <0.001 |

Adjusted for age and other cardiovascular risk diseases.
